# Occurrence of and risk factors for extended-spectrum cephalosporin-resistant *Enterobacteriaceae* determined by sampling of all Norwegian broiler flocks during a six month period

**DOI:** 10.1371/journal.pone.0223074

**Published:** 2019-09-26

**Authors:** Solveig Sølverød Mo, Anne Margrete Urdahl, Live Lingaas Nesse, Jannice Schau Slettemeås, Silje Nøstvedt Ramstad, Mona Torp, Madelaine Norström

**Affiliations:** 1 Department of Animal Health, Welfare and Food Safety, Norwegian Veterinary Institute, Oslo, Norway; 2 Department of Analysis and Diagnostics, Norwegian Veterinary Institute, Oslo, Norway; University of Georgia, UNITED STATES

## Abstract

All broiler flocks reared and slaughtered in Norway from May-October 2016 (n = 2110) were screened for the presence of extended-spectrum cephalosporin (ESC) -resistant *Enterobacteriaceae*. Furthermore, we investigated possible risk factors for occurrence of such bacteria in broiler flocks. The odds of a flock being positive for ESC-resistant *Enterobacteriaceae* increased if the previous flock in the same house was positive, and if the flock was reared during September-October. However, we cannot exclude seasonal fluctuations in occurrence of ESC-resistant *Enterobacteriaceae* during the months November to April. The overall occurrence of ESC-resistant *Enterobacteriaceae* was 10.4%, and primarily linked to the presence of *bla*_CMY_ (82.6%) in positive isolates. We describe the first findings of *Escherichia coli* with *bla*_CTX-M-1_, *Klebsiella pneumoniae* with both *bla*_CTX-M-15_ and *bla*_SHV-12_, and *K*. *pneumoniae* with *bla*_CMY_ isolated from Norwegian broiler production. This study gives us a unique overview and estimate of the true occurrence of ESC-resistant *Enterobacteriaceae* in Norwegian broilers over a six-month period. To the best of our knowledge, this is the most comprehensive study performed on the occurrence of ESC-resistant *Enterobacteriaceae* in a broiler population.

## Introduction

Since the first description of *Escherichia coli* (*E*. *coli*) from broilers displaying resistance towards extended-spectrum cephalosporins (ESC) in 2000–2001 [1], numerous studies have underlined the global distribution of ESC-resistant *Enterobacteriaceae* in broiler production [2–7]. In Norway, the first ESC-resistant *E*. *coli* isolated from food-producing animals was found in the intestinal flora of healthy broilers in 2006 [8, 9]. After implementation of a selective method for detection of ESC-resistant *E*. *coli* in the Norwegian monitoring programme on antimicrobial resistance in the veterinary sector (NORM-VET) in 2011, the occurrence has ranged between 6.3–42.9% in samples from parent flocks, broiler flocks and retail chicken meat [10–15]. Selection pressure from antimicrobial use is virtually absent in Norwegian broiler production, with between one and seven broiler flocks treated yearly from 2013–2017 [16–20]. Furthermore, cephalosporins are not used [12]. Imported parent/grandparent stocks have previously been identified as a potential source of ESC-resistant *E*. *coli* for the broiler production [8, 9]. In a previous study we identified risk factors for the occurrence of ESC-resistant *E*. *coli* in Norwegian broiler flocks [21]. The results showed that implementation of a high level of biosecurity, including strict disinfection routines, could minimize the odds of ESC-resistant *E*. *coli* occurring in the flock. Surprisingly, the ESC-status (i.e. ESC-resistant *E*. *coli* detected or not detected) of the parent flock(s) supplying the broiler flock was not associated with the ESC-status of the broiler flock. However, the results indicated that local recirculation of ESC-resistant *E*. *coli* between production cycles could occur in the broiler house [21]. This has also been suggested by others [22–26]. Inadequate hygiene has also been identified as a risk factor for occurrence of *Campylobacter* spp. in broiler flocks [27]. In Norway, the occurrence of *Campylobacter* spp. in broiler flocks is monitored yearly from May through October. This includes sampling of all broiler flocks that are slaughtered before 51 days of age to determine the *Campylobacter* status, i.e. *Campylobacter* spp. present or absent, prior to slaughter [28]. The data from the *Campylobacter* surveillance programme in 2016 was available for the present study.

We wanted to investigate whether there was an association between occurrence of *Campylobacter* spp. and ESC-resistant *Enterobacteriaceae* in Norwegian broiler flocks. Our hypothesis was that if inadequate hygiene affects both the occurrence of ESC-resistant *E*. *coli* and *Campylobacter* spp. in Norwegian broiler flocks, the same flocks could be positive for both ESC-resistant *Enterobacteriaceae* and *Campylobacter* spp. Furthermore, the occurrence of ESC-resistant *Enterobacteriaceae* and the genetic background for ESC resistance were determined.

## Material and methods

### Sampling and bacterial isolates

Boot swab- and dust samples from all broiler flocks slaughtered from May-October 2016 (n = 2213 samples from 2110 flocks) collected in the surveillance programme for *Salmonella* in live animals, eggs and meat in Norway 2016 [29] were included in the study. In brief, broiler flocks were sampled 10–19 days before slaughter. One pair of boot swabs and one dust cloth were collected from each flock. Most flocks were sampled once (n = 2019). A limited number of flocks were sampled two (n = 86), three (n = 6) or four times (n = 1).

Boot swabs and dust cloths were pooled and analysed as one sample. They were soaked in 225 mL buffered peptone water (BPW-ISO) and incubated at 37±1°C for 18±2 hours. Thereafter, 10 μL were spread on MacConkey agar (BD Difco, Beckton, Dickinson and Company, Le Pont de Claire, France) supplemented with 1 mg/L cefotaxime (Duchefa, Haarlem, The Netherlands) and incubated at 37±1°C overnight. For positive samples, one colony with typical morphology was re-plated on MacConkey agar supplemented with 1 mg/L cefotaxime and on blood agar, and the bacterial species was determined using matrix-assisted laser desorption/ionization time-of-flight (MALDI-TOF, Bruker Daltonics, GmbH, Bremen, Germany).

### Antimicrobial susceptibility testing

Minimum inhibitory concentrations (MICs) to a panel of antimicrobials were determined for all isolates (n = 229) by broth microdilution (EUVSEC and EUVSEC2, Sensititre, TREK Diagnostic LTD, Thermo Scientific). Susceptible *E*. *coli* ATCC25922 and ESC-resistant *E*. *coli* K5-20 (*bla*_CMY-2_) and K8-1 (*bla*_CTX-M-15_) were included as quality control in the susceptibility testing.

### Identification of resistance genes

Isolates displaying an AmpC-phenotype (i.e. resistance to cefoxitin, susceptible to cefepime and no synergy with clavulanic acid) were subjected to real-time PCR with previously published primers and probe [30] for detection of *bla*_CMY_. If the isolates were *bla*_CMY_ negative, they were subjected to PCR and sequencing for detection of plasmid-mediated AmpC (pAmpC) genes *bla*_MOX_, *bla*_CIT_, *bla*_DHA_, *bla*_ACC_, *bla*_EBC_ and *bla*_FOX_ [31], and PCR and sequencing for detection of up-regulated chromosomal AmpC production [32]. Isolates displaying an extended-spectrum beta-lactamase (ESBL) phenotype (i.e. resistant to cefepime, susceptible to cefoxitin and synergy with clavulanic acid) were subjected to PCR and sequencing using previously published primers for detection of *bla*_CTX-M_ [33], *bla*_TEM_ and *bla*_SHV_ [1] genes. Positive and negative controls were included in each PCR run. An overview of the positive controls included in the different PCR setups is included in S1 File.

### Data sources and data management

Data management and descriptive statistics were performed in SAS Enterprise Guide version 6.1 for Windows (SAS Institute Inc., Cary, NC, USA).

In addition to the data obtained through screening for the presence of ESC-resistant *Enterobacteriaceae* in the present study, data from the surveillance programme for *Campylobacter* spp. in broiler flocks in Norway in 2016 [28] were available from the Sample recording system at the Norwegian Veterinary Institute. These data included information regarding the FarmID, HouseID and FlockID in addition to sampling date and results from the real-time PCR for detection of *Campylobacter* spp. in the samples. The data were checked for misspelling and missing information (mainly HouseID) and harmonised to facilitate merging the data from the *Campylobacter* spp. surveillance programme and the present study by three levels of identification (ID); FarmID, HouseID and FlockID in order to study possible associations between the occurrence of ESC-resistant *Enterobacteriaceae* and *Campylobacter* spp. Most of the producers only had one house (n = 511), and in case of no houses recorded at any of the sample time points, the HouseID was coded as “HOUSE 1”.

In total, samples from 2110 broiler flocks originating from 583 producers and 701 broiler houses were analysed for the presence of ESC-resistant *Enterobacteriaceae* in this study. The results constitute the original dataset. The flocks were sampled at one (n = 2019), two (n = 86), three (n = 6) or four (n = 1) timepoints. When preparing datasets for univariable and multivariable analysis, only the first sample collected from each broiler flock was included. The ESC-status (i.e. *Enterobacteriaceae* with plasmid-mediated ESC resistance present or absent) of the first flock sampled in each broiler house (n = 701) was used to create the variable “ESC-status of previous broiler flock in the same house”. Thereafter, these first observations were excluded from the dataset so that all flocks included in the dataset had information regarding the ESC-status of the previous flock in the same house. Due to the hierarchical structure of the data, we included a nested random effect of broiler house within broiler farm. Thus, all producers with a single observation (n = 72) were excluded, as a minimum of two observations is required to include a nested random effect. This resulted in a dataset including results from 1307 flocks originating from 463 producers.

The original dataset was also merged with data obtained in the *Campylobacter* surveillance programme. In total, 1302 (62%) of the 2110 flocks included could be merged. We excluded the first observation from each house (n = 579) to allow inclusion of the variable “ESC-status of previous broiler flock in the same house”, resulting in a dataset including results from 723 flocks from 428 producers and 476 broiler houses. In addition to the variables described in the previous section, variables describing the *Campylobacter* status of the previous flock reared in the same house and the *Campylobacter* status at the farm were included (S4 Table).

### Univariable and multivariable analysis

Univariable and multivariable analyses were performed in R version 3.5.0 (R Foundation for Statistical Computing, Vienna, Austria).

Variables describing potential risk factors included in the univariable analyses are described in the supplementary material (S1–S3 Tables). First, all explanatory variables were fit into univariate logistic regression models using the glm function in R. The ESC status of the flock was used as the binary outcome. In addition, all variables in the original dataset were fit in separate univariable logistic regression models using the glmer function in the lme4 library in R. This was done to facilitate inclusion of a nested random effect of HouseID within FarmID or a random effect of FarmID. Variables were considered for inclusion in the multivariable analysis if the *p*-value was ≤ 0.20.

Associations between pairs of the included variables were tested using Pearson Chi-squared (two categorical variables). If a significant association was present, the most biologically plausible variable or the variable with the strongest association to the outcome was selected for inclusion in the multilevel model. The multilevel models were built by backward selection. A *p*-value of ≤ 0.05 was set as criterion for a variable to be retained in a model. As the data had a hierarchical structure, the modelling was performed both with and without a nested random effect of HouseID within FarmID and a random effect of FarmID for the original dataset. In the end, all previously excluded variables were tested against the model to examine whether any confounding effects were present. Models were compared using Akaike information criterion (AIC) and ANOVA. To assess the overall predictive ability of the model, we used a receiver-operating characteristics (ROC)-curve, and the area under the curve (AUC) was calculated. Residuals were plotted against predicted values and variables included in the model. The 95% confidence intervals (CIs) were calculated using estimated coefficients from the multivariable model. This was used to predict the strength of the association between each of the variables and the outcome.

## Results

### Descriptive results

#### Original dataset

*Enterobacteriaceae* with phenotypic cephalosporin resistance were isolated from 230 (10.4%) of the 2213 samples collected. The isolates were identified as *E*. *coli* (n = 228, 99.1%) or *K*. *pneumoniae* (n = 2, 0.9%). Most isolates (n = 202, 87.8%) displayed an AmpC phenotype and 190 isolates (82.6%) carried a *bla*_CMY_ gene. The remaining 12 isolates (5.2%) displayed up-regulated chromosomal *ampC* production due to mutations in the promoter region. In addition, 27 (11.7%) isolates displaying an ESBL phenotype were detected, of which all but one carried the *bla*_CTX-M-1_ gene. The last isolate (0.4%) carried both *bla*_CTX-M-15_ and *bla*_SHV-12_. In addition, a single isolate (0.4%) displaying phenotypic cephalosporin resistance was lost and therefore not subjected to genotyping. The two *K*. *pneumonia* isolates harboured *bla*_CTX-M-15_ and *bla*_CMY-2_, respectively. A median of three (range 1–5) broiler flocks were sampled from each house. In 38 houses (5.4%), more than one positive flock was detected. The same genetic background for ESC-resistance was present in all positive flocks in 31 (81.6%) of these houses, while different genetic background for ESC resistance was detected in flocks in seven (18.4%) houses. The number of houses at a farm ranged from one to eight houses with a median of one house, and 72 producers had more than one house at their farm.

#### Dataset including results from the *Campylobacter* surveillance programme

The 428 producers included had between one and five different houses (median one) and produced between one and three flocks from each house (median 2) during the sampling period. Of the 428 producers included, 35 (8.2%) had two or more houses at the farm.

*Campylobacter* spp. was detected in 70 of the 723 (9.7%) flocks included in the dataset. Furthermore, *Enterobacteriaceae* with plasmid-mediated ESC resistance were present in 48 (6.6%) of the included flocks. The occurrence of ESC-resistant *Enterobacteriaceae* in *Campylobacter* spp. positive flocks was 7.1% (n = 5), while it was 6.6% (n = 43) in *Campylobacter* negative flocks.

#### Antimicrobial susceptibility testing

MIC distributions for all *E*. *coli* with plasmid-mediated cephalosporin resistance (n = 215) are presented in Table 1. In total, 33 (15.3%) isolates displayed a multidrug resistant phenotype (i.e. resistant to ≥3 antimicrobial classes), while 145 (67.4%) isolates displayed beta-lactam resistance only. None of the isolates displayed resistance to colistin or carbapenems.

**Table 1:** Antimicrobial susceptibility testing. Minimum inhibitory concentrations (MICs) and antimicrobial resistance of extended-spectrum beta-lactamase-resistant *Escherichia coli* isolates (n = 215) with plasmid-mediated resistance mechanisms originating from Norwegian broiler flocks sampled during May-October 2016.

Substance	Resistance (%) [95% CI]		Distribution (%) of MIC values (mg/L)*															
			0.015	0.03	0.06	0.125	0.25	0.5	1	2	4	8	16	32	64	128	256	≥ 512
TET	15.3	[10.5–20.1]								84.2	0.5			0.5	13.5	1.4		
TIG	0	[0–1.6]					99.1	0.9										
CHL	0	[0–1.6]										99.5	0.5					
AMP	100	[97.8–100]													1.4	98.6		
CTX	100	[97.8–100]									4.2	95.8						
CAZ	99.5	[98.6–100]						0.5	5.6	6.0	0.9	55.3	31.6					
MER	0	[0–1.6]	100.0															
SUL	30.7	[24.5–36.9]										69.3						30.7
TRM	0.5	[0–1.2]					99.1	0.5						0.5				
AZI	ND								19.5	53.0	27.4							
GEN	16.3	[11.4–21.2]						75.8	7.4	0.5		0.9	12.1	2.8	0.5			
CIP	1.4	[0–2.8]	90.7	7.4	0.5	0.9	0.5											
NAL	1.4	[0–2.8]									98.6					0.5	0.9	
COL	0	[0–1.6]							100.0									

*Bold vertical lines denote epidemiological cut-off values for resistance. ND = cut-off not defined by EUCAST. CI = confidence interval. White fields denote range of dilutions tested for each antimicrobial agent. MIC values higher than the highest concentration tested are given as the lowest MIC value above the range. MIC values equal to or lower than the lowest concentration tested are given as the lowest concentration tested. TET = tetracycline, TIG = tigecycline, CHL = chloramphenicol, AMP = ampicillin, CTX = cefotaxime, CAZ = ceftazidime, MER = meropenem, SUL = sulfamethoxazole, TRM = trimethoprim, AZI = azithromycin, GEN = gentamicin, CIP = ciprofloxacin, NAL = nalidixic acid, COL = colistin

### Multivariable analysis

All results from the univariable analyses are shown in the supplementary material (S1–S4 Tables). The univariable models built from the dataset including data from the *Campylobacter* surveillance programme showed no significant association between the occurrence of ESC-resistant *Enterobacteriaceae* in broiler flocks and the occurrence of *Campylobacter* spp. in the same flock or at the farm during the study period (minimum one positive sample). An association was observed between the occurrence of ESC-resistant *Enterobacteriaceae* at the farm and the occurrence of *Campylobacter* spp. at the same farm (min two pos samples). However, this association was not significant in the multivariable model. Thus, we excluded data from the *Campylobacter* surveillance programme building the model using solely the original dataset in order to include as many observations as possible. The final three multivariable models (Table 2 and S5 and S6 Tables) built using the original dataset were compared by ANOVA, showing that both the random effect of FarmID (*p*<0,001) and the nested random effect of HouseID within FarmID (*p*<0.001) were significant. Thus, we chose the simplest model with the lowest AIC, namely the one including a random effect of FarmID. The model included the variables “Previous ESC-status”, and “Season” (Table 2). If the previous flock in the house was positive, the odds of the sampled flock being positive increased (OR = 3.1, 95% CI [1.4–6.8]. The same was observed for flocks sampled during September-October (OR = 10.0, 95% CI [2.3–43.4]) (Table 2). The area under the ROC curve was 0.90, indicating a good overall fit of the model to the observed data. The residual plots revealed no major deficiencies of the model.

**Table 2:** Risk factors for occurrence of ESC-resistant *Enterobacteriaceae* in Norwegian broilers. Results from the multivariable generalized model including a random effect of HouseID built to identify possible risk factors for occurrence of extended-spectrum cephalosporin-resistant *Enterobacteriaceae* in Norwegian broiler flocks (n = 1307) sampled from May-October 2016.

Variable		Estimate	SE	OR [95% CI]	*p*-value
Status of previous flock in the same house					
	Neg	0			
	Pos	1.1	0.4	3.1 [1.4–6.8]	0.006
Season					
	1 (May-June)	0			
	2 (July—August)	1.0	0.8	2.6 [0.6–11.7]	0.20
	3 (September-October)	2.3	0.7	10.0 [2.3–43.4]	0.002

AIC = 873.9. SE: standard error, OR: odds ratio, CI: confidence interval

## Discussion

In this study, we assessed possible risk factors for occurrence of ESC-resistant *Enterobacteriaceae* in Norwegian broiler flocks using the census population of all broiler flocks reared during the study period from May-October 2016. To the best of our knowledge, this is the most comprehensive study performed on the occurrence of ESC-resistant *Enterobacteriaceae* in broiler flocks and possible risk factors associated with such resistance. The odds of a flock being positive increased if the previous flock in the same house was positive (OR = 3.1), as we have also demonstrated previously [21]. This suggests a possible on-farm persistence of ESC-resistant *Enterobacteriaceae* between production rounds.

Our results also indicate that flocks sampled during September-October had a higher odds of being ESC-positive (OR = 10.0) compared to flocks sampled during May-August. This seasonal effect was not detected in our previous study [21], but has been described for *Campylobacter* spp. with the highest incidence of prevalence occurring between July and August [34, 35]. The observed effect can be due to climatic conditions, such as precipitation or temperature as described for *Campylobacter* spp. [36]. However, as we only collected samples during a six month period, we cannot rule out that the occurrence of ESC-resistant *Enterobacteriaceae* fluctuates during the winter season as well. Furthermore, a random effect of the farm was significantly associated with the ESC status, indicating that farm-specific factors not included in our variables have an impact on the occurrence of ESC-resistant *Enterobacteriaceae* in broiler flocks. We cannot exclude that other factors not investigated in this study can have significant impact on the occurrence and persistence of ESC-resistant *Enterobacteriaceae*. For example, sufficient disinfection routines were shown to lower the odds of detecting ESC-resistant *Enterobacteriaceae* in broilers in a previous study [21]. However, other factors such as floor type, litter type, empty days between production rounds, and biosecurity measures and routines among others, could also be of importance. Although we could not find any significant association between ESC-status and *Campylobacter* status of the flock or the farm, farm-associated factors relating to hygiene cannot be ruled out. *Campylobacter* spp. is ubiquitous in the environment, and inadequate biosecurity is a well known source for transmission to poultry [34, 37, 38]. Introduction of ESC-resistant *Enterobacteriaceae* is mediated by broilers carrying the bacteria and/or cross-contamination from previous flocks. Therefore, an adequate hygienic barrier is important in order to prevent introduction of *Campylobacter* spp. However, if first introduced, inadequate in-house hygiene may facilitate maintenance of both ESC-resistant *Enterobacteriaceae* and *Campylobacter* spp.

The genetic background for ESC-resistance in broiler production in most other European countries is complex, including both AmpC- and ESBL-encoding genes [3, 5, 39–43]. In contrast, all ESC-resistant *E*. *coli* collected from Norwegian broiler production, with the exception of the first isolate detected in 2006 [8, 9], have displayed an AmpC phenotype. The genetic background for the resistant phenotype has been *bla*_CMY_ or an up-regulated chromosomal *ampC* production [10–14, 21]. In the present study however, we describe the emergence of the ESBL-encoding gene *bla*_CTX-M-1_ in the Norwegian broiler production. In addition, we detected single isolates carrying *bla*_CTX-M-15_ and *bla*_SHV-12_. Both *bla*_CTX-M-1_ and *bla*_SHV-12_ genotypes have previously been reported from broiler production in Sweden and Denmark [22, 44–46], and are also common in other European countries [3, 5]. As parent animals and broilers in Sweden, Denmark and Norway originate from the same grandparent animals [21, 22], the emerging genotypes may have been introduced via contaminated parent and/or grandparent stocks, as previously shown for pAmpC-producing *E*. *coli* [8, 9, 22, 26].

Since the start of 2014, the Norwegian broiler industry has tested all batches of hatching eggs imported to Norway for the presence of ESC-resistant *E*. *coli*. In 2015, only 2.4% of the imports (n = 84) were ESC positive [17]. These imports were probably the origin of most flocks sampled in the present study, thus indicating a limited transmission of ESC-resistant *Enterobacteriaceae* from parent to broilers. Unfortunately, there is no information available regarding the genetic background of the ESC-resistant *E*. *coli* found in the positive imports, and we can therefore not be sure that the emerging genotypes were present in newly hatched parent animals. Nor can we exclude that *Enterobacteriaceae* with *bla*_CTX-M-1_, *bla*_CTX-M-15_ and *bla*_SHV-12_ have been present in the Norwegian broiler production at a previous stage. Only a limited number of samples are investigated in the NORM-VET programme, and only a single ESC-resistant isolate is characterized per positive sample. Our results show that the *bla*_CMY-2_ genotype is by far the most common genotype among the ESC-resistant isolates. The low occurrence of the *bla*_CTX-M_ and *bla*_SHV_ genotypes require investigation of a high number of samples in order to detect a positive sample. Thus, it is possible that the presence of the “new” genotypes have been masked by the more prevalent *bla*_CMY-2_ in samples investigated in the NORM-VET programme. We detected ESC-producing *K*. *pneumonia* in two of the samples, representing two different broiler flocks. This represents the first description of ESC-resistant *K*. *pneumonia* in Norwegian broiler production. However, we did not use agar selective for *Klebsiella* spp., and this has not been done in the NORM-VET programme either. Thus, we cannot exclude that there may have been an undetected reservoir of ESC-resistant *Klebsiella* spp. in the broiler production chain. Further studies using selective methods targeting *Klebsiella* spp. may be warranted.

The data from this study gives us a unique overview of the situation regarding occurrence of ESC-resistant *Enterobacteriaceae* in Norwegian broiler flocks in 2016 as all flocks reared from May to October were sampled. To the best of our knowledge, such an extensive study has not been performed previously. It gives us a precise estimate of the true occurrence of ESC-resistant *Enterobacteriaceae* in Norwegian broilers over the six month study period. Also, the long study period gave us the opportunity to sample consecutive flocks in the same houses. This approach enabled us to consider the possibility of persistence of ESC-resistant strains in houses between production cycles. The same ESC resistance genotype was detected in at least two flocks in 33 houses, indicating a possible persistence of ESC-resistant *E*. *coli* in the broiler house between production cycles. However, different genotypes were detected in positive flocks in seven houses. This may be due to the presence of several ESC-resistance genotypes in the house, but may also be explained by new introduction of ESC-resistant *E*. *coli* from supplying parent flocks. Data on the occurrence of ESC-resistant *E*. *coli* in the supplying parent flocks was not available for the current study, and further investigations on this matter could therefore not be performed.

The overall occurrence of ESC-resistant *Enterobacteriaceae* was 10.4% of the samples. In total, two *K*. *pneumoniae* isolates were detected, while the rest of the ESC-resistant isolates were identified as *E*. *coli*. Due to small differences in the sample material investigated and detection methods used, it is not possible to compare these results directly with results from the NORM-VET programme. However, the results indicate a significant reduction in the occurrence of ESC-resistant *E*. *coli* in Norwegian broilers since 2011, as has also been seen in the NORM-VET programme [14]. The same trend has also been reported in Sweden [46] and Denmark [44]. Moreover, the Norwegian broiler industry has recently reported that all batches of imported hatching eggs have been negative since the end of 2016 [18, 19]. This is also reflected in the results from the NORM-VET programme in 2018 where only a single positive sample from broilers was found (NORM-NORM/VET 2018, submitted for publication).

In conclusion, we have identified that a positive ESC status of the previous broiler flock and rearing during September-October increases the odds for detecting ESC-resistant *Enterobacteriaceae* in broiler flocks. There was no association between the occurrence of *Campylobacter* spp. and ESC-resistant *Enterobacteriaceae* in broiler flocks. This is the first study including sampling of the census broiler population over a prolonged period, and the first description of the ESC-resistance genotypes *bla*_CTX-M-1_, *bla*_CTX-M-15_ and *bla*_SHV-12_ in *Enterobacteriaceae* isolated from broilers in Norway.

## Supporting information

S1 Table Univariable analysis. Results from the univariable analysis on potential risk factors for occurrence of extended-spectrum beta-lactamase-producing *Enterobacteriaceae* in Norwegian broiler flocks (n = 1307) sampled from May- October 2016. Overall *p*-values for variables with more than two categories were calculated using the likelihood ratio test.
(DOCX)

S2 Table Univariable analysis including random effect of farm. Results from the univariable analysis on potential risk factors for occurrence of extended-spectrum beta-lactamase-producing *Enterobacteriaceae* in Norwegian broiler flocks (n = 1307) sampled from May- October 2016 including a random effect of farm. Overall *p*-values for variables with more than two categories were calculated using the likelihood ratio test.
(DOCX)

S3 Table Univariable analysis including nested random effect of house within farm. Results from the univariable analysis on potential risk factors for occurrence of extended-spectrum beta-lactamase-producing *Enterobacteriaceae* in Norwegian broiler flocks (n = 1307) sampled from May- October 2016 including a nested random effect of house within farm. Overall *p*-values for variables with more than two categories were calculated using the likelihood ratio test.
(DOCX)

S4 Table Univariable analysis including results from the *Campylobacter* spp. surveillance programme. Results from the univariable analysis on potential risk factors for occurrence of extended-spectrum beta-lactamase-producing *Enterobacteriaceae* in Norwegian broiler flocks sampled from May- October 2016. The dataset used for analysis contain data from 723 flocks on 428 farms, including data from the *Campylobacter* surveillance programme. Overall *p*-values for variables with more than two categories were calculated using the likelihood ratio test.
(DOCX)

S5 Table Multivariable model without random effect. Results from the multivariable generalized model built to identify possible risk factors for occurrence of extended-spectrum cephalosporin-resistant *Enterobacteriaceae* in Norwegian broiler flocks (n = 1307) sampled from May-October 2016.
(DOCX)

S6 Table Multivariable model including nested random effect of house within farm. Results from the multivariable generalized model including a nested random effect of HouseID within FarmID built to identify possible risk factors for occurrence of extended-spectrum cephalosporin-resistant *Enterobacteriaceae* in Norwegian broiler flocks (n = 1307) sampled from May-October 2016.
(DOCX)

S1 File PCR controls. Overview of positive and negative controls included in the different PCR setups for detection of genes encoding Extended-spectrum cephalosporin resistance.
(DOCX)
