# Supplementary material for: Occurrence of and risk factors for extended-spectrum cephalosporin-resistant Enterobacteriaceae determined by sampling of all Norwegian broiler flocks during a six month period
Source: PLoS One. 2019 Sep 26;14(9):e0223074. doi: 10.1371/journal.pone.0223074 (PMC6762140; doi:10.1371/journal.pone.0223074)
Supplement: S1 Table — Results from the univariable analysis on potential risk factors for occurrence of extended-spectrum beta-lactamase-producing Enterobacteriaceae in Norwegian broiler flocks (n = 1307) sampled from May- October 2016. Overall p-values for variables with more than two categories were calculated using the likelihood ratio test. (DOCX) [file pone.0223074.s001.docx]

**S1 Table. Univariable analysis.** Results from the univariable analysis on potential risk factors for occurrence of extended-spectrum beta-lactamase-producing *Enterobacteriaceae* in Norwegian broiler flocks (n=1307) sampled from May- October 2016. Overall *p*-values for variables with more than two categories were calculated using the likelihood ratio test.

| **Variable** | | **Negative flocks (No.)** | **Positive flocks (No.)** | **OR [95% CI]** | ***p*-value** | **AIC** | **Overall *p*-value** |
| --- | --- | --- | --- | --- | --- | --- | --- |
| ESC status of previous flock in the same house | |  |  |  |  | 927.3 |  |
|  | Neg | 1100 | 127 |  |  |  |  |
|  | Pos | 49 | 31 | 5.5 [3.4-8.9] | <0.001 |  |  |
| Season |  |  |  |  |  | 915.0 | <0.001 |
|  | 1 (May-June) | 74 | 2 |  |  |  |  |
|  | 2 (July-August) | 580 | 40 | 2.6 [0.6-10.8] | 0.20 |  |  |
|  | 3 (September-October) | 495 | 116 | 8.7 [2.1-35.8] | 0.003 |  |  |
| Geography | |  |  |  |  | 967.7 | 0.36 |
|  | East | 502 | 60 |  |  |  |  |
|  | Midd | 335 | 53 | 1.3 [0.9-2.0] | 0.16 |  |  |
|  | West | 312 | 45 | 1.2 [0.8-1.8] | 0.37 |  |  |
| Number of houses at farm | |  |  |  |  | 966.8 |  |
|  | 1 | 919 | 121 |  |  |  |  |
|  | >1 | 230 | 37 | 1.2 [0.8-1.8] | 0.32 |  |  |
| Number of flocks in house during sampling period (factor) | | |  |  |  | 963.1 | 0.04 |
|  | 2 | 491 | 51 |  |  |  |  |
|  | 3 | 618 | 99 | 1.5 [1.1-2.2] | 0.02 |  |  |
|  | 4 | 40 | 8 | 1.9 [0.9-4.3] | 0.11 |  |  |

Null model: AIC=965.8. OR: odds ratio, CI: confidence interval
