# Supplementary material for: Occurrence of and risk factors for extended-spectrum cephalosporin-resistant Enterobacteriaceae determined by sampling of all Norwegian broiler flocks during a six month period
Source: PLoS One. 2019 Sep 26;14(9):e0223074. doi: 10.1371/journal.pone.0223074 (PMC6762140; doi:10.1371/journal.pone.0223074)
Supplement: S2 Table — Results from the univariable analysis on potential risk factors for occurrence of extended-spectrum beta-lactamase-producing Enterobacteriaceae in Norwegian broiler flocks (n = 1307) sampled from May- October 2016 including a random effect of farm. Overall p-values for variables with more than two categories were calculated using the likelihood ratio test. (DOCX) [file pone.0223074.s002.docx]

**S2 Table. Univariable analysis including random effect of farm.** Results from the univariable analysis on potential risk factors for occurrence of extended-spectrum beta-lactamase-producing *Enterobacteriaceae* in Norwegian broiler flocks (n=1307) sampled from May- October 2016 including a random effect of farm. Overall *p*-values for variables with more than two categories were calculated using the likelihood ratio test.

| **Variable** | | **Negative flocks (No.)** | **Positive flocks (No.)** | **OR [95% CI]** | ***p*-value** | **AIC** | **Overall *p*-value** |
| --- | --- | --- | --- | --- | --- | --- | --- |
| ESC status of previous flock in the same house | |  |  |  |  | 924.7 |  |
|  | Neg | 1100 | 127 |  |  |  |  |
|  | Pos | 49 | 31 | 3.8 [1.9-7.3] | <0.001 |  |  |
| Season |  |  |  |  |  | 887.9 | <0.001 |
|  | 1 (May-June) | 74 | 2 |  |  |  |  |
|  | 2 (July-August) | 580 | 40 | 2.6 [0.6-11.7] | 0.22 |  |  |
|  | 3 (September-October) | 495 | 116 | 11.5 [2.5-50.0] | 0.002 |  |  |
| Geography | |  |  |  |  | 937.1 | 0.49 |
|  | East | 502 | 60 |  |  |  |  |
|  | Midd | 335 | 53 | 1.3 [0.8-2.2] | 0.31 |  |  |
|  | West | 312 | 45 | 1.3 [0.8-2.3] | 0.32 |  |  |
| Number of houses at farm | |  |  |  |  | 936.3 |  |
|  | 1 | 919 | 121 |  |  |  |  |
|  | >1 | 230 | 37 | 1.1 [0.6-2.1] | 0.68 |  |  |
| Number of flocks in house during sampling period (factor) | | |  |  |  | 932.5 | 0.05 |
|  | 2 | 491 | 51 |  |  |  |  |
|  | 3 | 618 | 99 | 1.7 [1.1-2.8] | 0.03 |  |  |
|  | 4 | 40 | 8 | 2.4 [0.7-7.8] | 0.15 |  |  |

Null model: AIC=934.5. OR: odds ratio, CI: confidence interval
