# Supplementary material for: Occurrence of and risk factors for extended-spectrum cephalosporin-resistant Enterobacteriaceae determined by sampling of all Norwegian broiler flocks during a six month period
Source: PLoS One. 2019 Sep 26;14(9):e0223074. doi: 10.1371/journal.pone.0223074 (PMC6762140; doi:10.1371/journal.pone.0223074)
Supplement: S4 Table — Results from the univariable analysis on potential risk factors for occurrence of extended-spectrum beta-lactamase-producing Enterobacteriaceae in Norwegian broiler flocks sampled from May- October 2016. The dataset used for analysis contain data from 723 flocks on 428 farms, including data from the Campylobacter surveillance programme. Overall p-values for variables with more than two categories were calculated using the likelihood ratio test. (DOCX) [file pone.0223074.s004.docx]

**S4 Table.** **Univariable analysis including results from the *Campylobacter* spp. surveillance programme.** Results from the univariable analysis on potential risk factors for occurrence of extended-spectrum beta-lactamase-producing *Enterobacteriaceae* in Norwegian broiler flocks sampled from May- October 2016. The dataset used for analysis contain data from 723 flocks on 428 farms, including data from the *Campylobacter* surveillance programme. Overall *p*-values for variables with more than two categories were calculated using the likelihood ratio test.

| **Variable** | | **Negative flocks (No.)** | **Positive flocks (No.)** | **OR [95% CI]** | ***p*-value** | **AIC** | **Overall *p*-value** |
| --- | --- | --- | --- | --- | --- | --- | --- |
| ESC status of previous flock in the same house | |  |  |  |  | 325.3 |  |
|  | Neg | 650 | 34 |  |  |  |  |
|  | Pos | 25 | 14 | 10.7 [5.1-22.4] | <0.001 |  |  |
| Season |  |  |  |  |  | 354.4 | 0.10 |
|  | 1 (May-June) | 58 | 1 |  |  |  |  |
|  | 2 (July-August) | 466 | 32 | 4.0 [0.5-29.7] | 0.18 |  |  |
|  | 3 (September-October) | 151 | 15 | 5.8 [0.7-44.6] | 0.09 |  |  |
| Geography | |  |  |  |  | 344.7 | <0.001 |
|  | East | 298 | 16 |  |  |  |  |
|  | Midd | 190 | 26 | 2.5 (1.3-4.9] | 0.005 |  |  |
|  | West | 187 | 6 | 0.6 (0.2-1.6] | 0.29 |  |  |
| *Campylobacter* present in the same flock | |  |  |  |  | 357.1 |  |
|  | No | 610 | 43 |  |  |  |  |
|  | Yes | 65 | 5 | 1.1 [0.4-2.9] | 0.86 |  |  |
| *Campylobacter* status at farm (min one pos sample) | | |  |  |  | 356.7 |  |
|  | Neg | 568 | 42 |  |  |  |  |
|  | Pos | 107 | 6 | 0.8 [0.3-1.8] | 0.54 |  |  |
| *Campylobacter* status at farm (min two pos sample) | | |  |  |  | 354.4 |  |
|  | Neg | 669 | 46 |  |  |  |  |
|  | Pos | 6 | 2 | 4.9 [1.0-24.7] | 0.06 |  |  |
| Number of houses at farm | |  |  |  |  | 354.5 |  |
|  | 1 | 558 | 35 |  |  |  |  |
|  | >1 | 117 | 13 | 1.8 [0.9-3.5] | 0.09 |  |  |
| Number of flocks in house during sampling period | | |  |  |  | 352.3 | 0.03 |
|  | 1 | 230 | 12 |  |  |  |  |
|  | 2 | 413 | 29 | 1.3 [0.7-2.7] | 0.04 |  |  |
|  | 3 | 32 | 7 | 4.2 [1.5-11.4] | 0.005 |  |  |

Null model: AIC=355.1. OR: odds ratio, CI: confidence interval
