# Supplementary material for: Occurrence of and risk factors for extended-spectrum cephalosporin-resistant Enterobacteriaceae determined by sampling of all Norwegian broiler flocks during a six month period
Source: PLoS One. 2019 Sep 26;14(9):e0223074. doi: 10.1371/journal.pone.0223074 (PMC6762140; doi:10.1371/journal.pone.0223074)
Supplement: S6 Table — Results from the multivariable generalized model including a nested random effect of HouseID within FarmID built to identify possible risk factors for occurrence of extended-spectrum cephalosporin-resistant Enterobacteriaceae in Norwegian broiler flocks (n = 1307) sampled from May-October 2016. (DOCX) [file pone.0223074.s006.docx]

**S6 Table.** **Multivariable model including nested random effect of house within farm.** Results from the multivariable generalized model including a nested random effect of HouseID within FarmID built to identify possible risk factors for occurrence of extended-spectrum cephalosporin-resistant *Enterobacteriaceae* in Norwegian broiler flocks (n=1307) sampled from May-October 2016.

| **Variable** |  | **Estimate** | **SE** | **OR [95% CI]** | ***p*-value** |
| --- | --- | --- | --- | --- | --- |
| Status of previous flock in the same house | |  |  |  |  |
|  | Neg | 0 |  |  |  |
|  | Pos | 1.1 | 0.4 | 3.0 [1.4-6.8] | 0.006 |
| Season |  |  |  |  |  |
|  | 1 (May-June) | 0 |  |  |  |
|  | 2 (July - August) | 1.0 | 0.8 | 2.6 [0.6-11.7] | 0.20 |
|  | 3 (September-October) | 2.3 | 0.7 | 10.0 [2.3-43.4] | 0.002 |

AIC=875.9. SE: standard error, CI: confidence interval
