# Supplementary material for: Occurrence of and risk factors for extended-spectrum cephalosporin-resistant Enterobacteriaceae determined by sampling of all Norwegian broiler flocks during a six month period
Source: PLoS One. 2019 Sep 26;14(9):e0223074. doi: 10.1371/journal.pone.0223074 (PMC6762140; doi:10.1371/journal.pone.0223074)
Supplement: S1 File — Overview of positive and negative controls included in the different PCR setups for detection of genes encoding Extended-spectrum cephalosporin resistance. (DOCX) [file pone.0223074.s007.docx]

Positive control included in real-time PCR for detection of *bla*_CMY_:

E. coli K5-20, *bla*_CMY-2_ positive

Positive controls included in the multiplex PCR for detection of plasmid-mediated ampC genes:

E. coli A4-27, *bla*_CMY-1_ positive (MOX)

E. coli K5-20, *bla*_CMY-2_ positive

E. coli 2009-04-20410, *bla*_DHA_ positive

E. coli A2-60, *bla*_ACC_ positive

E. coli A2-58, *bla*_EBC_ positive

E. coli A2-59, *bla*_FOX_ positive

Positive controls included in multiplex PCR for detection of ESBL genes:

*E. coli* K8-1, *bla*_CTX-M-15_ and *bla*_TEM_ positive

*Klebsiella pneumonia* ATCC 700603/CCUG 45421, *bla*_SHV_ positive

Negative controls included in all PCRs:

Mastermix with milliQ water added instead of DNA
